# Charcot arthropathy of the diabetic foot in a sub-Saharan tertiary hospital: a cross-sectional study

**DOI:** 10.1186/s13047-019-0343-0

**Published:** 2019-06-14

**Authors:** Jean Paul Vwakya Wanzou, Patrick Sekimpi, Johnson Owonda Komagum, Frederick Nakwagala, Erisa Sabakaki Mwaka

**Affiliations:** 1 Department of Orthopaedics, College of Health Sciences, Makerere University, P.O BOX 7072, Kampala, Uganda; 2 Child and Family Foundation, Kampala, Uganda; 3 Department of Internal medicine, Endocrinology Unit, Mulago Hospital, P.O BOX 7051, Kampala, Uganda; 4 Department of Anatomy, College of Health Sciences, Makerere University, P.O BOX 7072, Kampala, Uganda

**Keywords:** Charcot foot arthropathy, Diabetic foot, Sub-Saharan

## Abstract

**Background:**

Charcot foot arthropathy is a potentially limb-threatening condition that leads to progressive destruction of the bones and joints in the neuropathic foot. One of its main causes is diabetes mellitus whose prevalence is steadily increasing. The acute phase is often misdiagnosed thus leading to foot deformity, ulceration and increased risk of amputation. There is a paucity of literature on this condition from sub-Saharan Africa. This study aimed at determining the extent of Charcot foot arthropathy, the radiological patterns of Charcot foot arthropathy and patient’s factors associated with Charcot foot arthropathy among adult patients with longstanding diabetes in an African setting.

**Methods:**

This was a cross-sectional study that was carried at a national referral and university teaching hospital in Kampala, Uganda. One hundred patients with longstanding diabetes mellitus were consecutively recruited. Patients with a history of having diabetes mellitus for at least seven years since diagnosis were considered to have a longstanding disease. Clinical assessment of both feet was done. Weight-bearing radiographs of the selected foot were taken and evaluated using the Sanders and Frykberg and modified Eichenholtz classifications. A blood sample was taken for glycosylated haemoglobin (HbA1c). Data were summarized using descriptive statistics and student t-test.

**Results:**

The proportion of Charcot foot arthropathy among patients with longstanding diabetes was 12% of which one-third (4 out of 12) were acute cases. Fifty percent of the lesions were in the forefoot and 50% in the midfoot. Seventeen percent of lesions were at the inflammatory stage of the modified Eichenholtz classification, 50% at the developmental stage, 25% at the healing stage, and 8% at the remodelling stage. An abnormal foot radiograph was significantly associated with Charcot foot arthropathy among patients with longstanding diabetes.

**Conclusion:**

Charcot foot arthropathy is fairly common in patients with longstanding diabetes mellitus in these settings with one third of patients presenting in the early acute phase. An abnormal weight-bearing radiograph was an associated factor of Charcot foot arthropathy among this specific group of patients. To reduce on the morbidity and limb threatening sequelae of this condition, clinicians are therefore advised to routinely examine the feet of patients with diabetes and send those with suspicious signs and symptoms for radiographic assessment.

## Background

Diabetes mellitus is the most prevalent non-communicable chronic disease worldwide [1], with Africa having the highest prevalence of undiagnosed diabetes [2]. The prevalence of diabetes mellitus in Uganda is on the increase and it is becoming a major public health problem that is being compounded by limited access to quality health care [3–6]. Population-based studies in Uganda have reported diabetes prevalence rates ranging from 1.4 to 7.4% [3, 7]. Diabetes mellitus predisposes patients to foot complications that range from ulceration to gangrene that is associated with significant long-term disability and premature mortality [1]. Complication rates for foot complications in Africa vary from 4 to 19%; peripheral neuropathy, 4–84.4%; peripheral vascular disease, 2.9–78.7%; gangrene, 0.6–69%; and amputation rates of 0.3–45% [1]. However, very few studies from Africa have specifically addressed Charcot foot arthropathy (CA).

Charcot foot arthropathy (CA), is a condition affecting the bones and joints of the foot in patients with peripheral neuropathy of various origins, characterized in the earliest phase by non-infective inflammation of bones and joints of the foot [8–10] and in the later phases by progressive destruction of bones and joints in the weight-bearing neuropathic foot leading to acute fractures, dislocations and joint destruction [11, 12]. There are many reported aetiologies of CA, however, nowadays diabetes mellitus has become the leading cause [8, 12–15].

Globally, several population-based studies have reported an estimated CA prevalence ranging from 0.08% of the general diabetes patient population to 13% of patients presenting at diabetic foot clinics [12, 14, 16, 17] though the exact prevalence of CA is unknown. This might partly be attributed to a high incidence of mistaken or delayed initial diagnosis [12, 14, 16]. Charcot foot arthropathy is associated with several complications [18, 19] yet there is a paucity of literature on CA from sub-Saharan Africa. We came across only one study from East Africa that was conducted in a Kenyan district hospital and reported foot complication rates as high as 29.2% in patients attending a diabetic clinic; with 95% of these having diabetic ulcers and 5% had CA or cellulitis [20]. To the best of the author’s knowledge, no study related to CA in Uganda has yet been published.

The acute phase of CA is often misdiagnosed and can lead to permanent foot deformity, ulceration thus increasing the risk of lower extremity amputation [8, 12, 21, 22]. Early diagnosis and management of acute CA are therefore imperative to avoid progression towards permanent foot deformity and its associated complications [12, 13]. In patients with diabetes and an established CA, patients can present with foot ulceration secondary to foot deformity and therapeutic efforts tend to be focused on ulcers management rather than arresting the disease process [23].

There are 3 types of Charcot foot classification: clinical, anatomical and radiological [11, 12, 17, 24–26]. In clinical practice, Charcot foot can be classified into the acute and chronic stage [25]:

In the acute stage, the foot is remarkably red, warm and swollen; the midfoot is most affected and pain is not a prominent feature [25]. In the Chronic stage signs of local inflammation progressively recede [25, 27]; stable deformities may develop in this stage most commonly a rocker bottom deformity [25, 27]. The most frequently used anatomical classification was proposed by Sanders and Frykberg [11, 12, 17, 24–26]. It describes five different patterns of destruction. The radiological classification is based on the natural history of the disease and is divided into four stages based on Eichenholtz’s work in 1966, and later modified in 1990 by Shibata et al. [17, 24–28].

Potential risk factors associated with CA include age, duration of diabetes, body mass index, history of an instigating event such as foot trauma or foot surgery and peripheral neuropathy [11, 12, 14, 29, 30]. In patients with diabetes, CA typically presents during the fifth or sixth decade of life [31]. A longstanding history, at least a decade with diabetes is usual [32–34]. Most commonly, at the time of onset patients with both Type 1 and Type 2 diabetes have been diagnosed for a period ≥10 years, however, for type 2 other studies found a diabetes duration of 5 to 9 years at the time of CA diagnosis [11, 12, 14, 29, 30]. The mean body mass index of Charcot patients, according to Pakarinen et al. [32] was 32.9 kg/m^2^ and 34.5 kg/m^2^ in men and women, respectively. A history of an instigating event (foot trauma, foot surgery) preceding the onset of Charcot's foot has been reported from 22 to 73% of the time [35]. Peripheral neuropathy is associated with all disorders that produce neuroarthropathy and is believed to be the prerequisite for the development of Charcot arthropathy, however not all patients with diabetes and peripheral neuropathy will develop Charcot foot arthropathy [8, 14, 17, 29, 30].

The prevalence of diabetes mellitus is currently increasing worldwide including Uganda, becoming, therefore, a major public health problem [3–5]. If not managed in time, CA leads to permanent foot deformities, which will progress to foot ulcers, thus increasing the risk for lower extremity amputation. Charcot foot arthropathy is a contributing factor in the 15 to 40-fold increase in the risk of lower-extremity amputations in the population with diabetes [36]. Screening for diabetic complications in Uganda urban diabetic clinics is suboptimal [37]. Among patients with diabetes, peripheral neuropathy, the main risk factor of CA estimated to have a prevalence of 9 to 32% among patients with diabetes [14, 38, 39], was found to be 46.4% in Uganda at the time of diagnosis [5].

Despite the facts mentioned above, there was no baseline data in terms of extent and patterns of that foot disease in these clinics, there were no established protocol for its early diagnosis and management. Charcot foot arthropathy is overlooked and misdiagnosed; hence the increased morbidity increased risk of foot amputations and high economic burden among these patients.

Therefore, this study aimed at determining the extent of CA, the radiological patterns of CA and patient’s factors associated with CA among adult patients with longstanding diabetes attending MNRH.

## Methods

### Study design

This was a cross-sectional study that was conducted from June 2017 to April 2018.

### Study site

The study was carried out in the weekly diabetic outpatient clinic at Mulago National Referral Hospital (MNRH). Mulago hospital, located in the Ugandan capital city Kampala, is the largest national referral hospital in the country and is also the teaching hospital for Makerere University.

### Study participants

Participants included adult patients (at least 18 years) with long-standing diabetes mellitus who gave written informed consent. For this study, patients with a history of having diabetes mellitus for at least seven years since diagnosis were considered to have a longstanding disease. Patients with gangrene of the foot were excluded. Participants were consecutively recruited from a diabetic outpatient clinic. The sample size was 100 patients. We used an assumed prevalence of 7% based on Frykberg et al. [14] study that reported a CA prevalence of 0.08–13% and a precision of 5% (delta). To allow for adequate power, a confidence level of 95% was selected.

### Data collection

Adult patients with long-standing diabetes were consented and consecutively enrolled in the study. Data collected on history included demographic characteristics, duration since diagnosis of diabetes mellitus, and previous foot surgery or foot trauma. Clinical assessment was done for both feet after which one foot, the most affected, was selected for a weight-bearing foot radiographs. The feet were examined for swelling, deformity, ulceration, peripheral neuropathy and warmth. Foot ulcers were examined to rule out osteomyelitis which was clinically suspected if the ulcer had been present for more than one week, the lesion extending more than 2cm^2^, and a positive probe to bone test. The probe to bone test was considered positive if the bone could be felt using a sterile blunt metallic probe through the ulcer [9, 40].

Peripheral neuropathy was assessed using the 5.07/10 g Semmes-Weinstein monofilament to test for sensation on the plantar aspect of the first, third, and fifth metatarsal heads. The monofilament was applied to the test site perpendicularly until it bent for about one second. The patients were instructed to say “yes” each time they sensed the monofilament. If the patient failed to sense the monofilament after bending, the test site was considered to be insensate. Patients unable to detect touch at one or more sites were considered to have abnormal sensation. Only patients able to feel all the examination sites were considered to have a normal sensation. [41–45]. The warmth was assessed using a handheld infrared dermal thermometer (Elektro™ Orb Genesis LLC, Istanbul, Turkey). The temperature was taken on the dorsal aspect of the midfoot or the most prominent site of the swelling. A difference of more than 2 °C between the two feet implied the presence of local inflammation, raising suspicion of the acute phase CA.

Radiographs were interpreted using Sanders and Frykberg anatomical classification [11, 12, 17, 24–26] and the modified Eichenholtz classification [17, 24–28].

Sanders and Frykberg anatomical classification:

**Table:** 

Type / Pattern	Anatomic location
Pattern I	Forefoot (metatarsophalangeal and interphalangeal joints)
Pattern II	Tarso-metatarsal joints
Pattern III	Talonavicular, naviculocuneiform, and calcaneocuboid joints
Pattern IV	Ankle and subtalar joints
Pattern V	Calcaneum

Modified Eichenholtz classification:

**Table:** 

Stage	Clinical findings	Radiological findings
0 Inflammatory	Localized warmth, oedema/swelling and erythema	Almost normal or minimal abnormality
1 Developmental	Marked localized swelling, warmth, and redness; minor bone deformity, joint instability (ligamentous laxity).	Focal bone demineralization (osteopenia). Bony debris at articular margins. Fragmentation of subchondral bone. Periarticular fracture. Subluxation, and/or dislocation.
2 Healing	Continued but decreased warmth, oedema and erythema, major bone deformity, bone instability	Absorption of fine osseous debris. Coalescence/fusion of bone fragments. Callus formation and/or new periosteal bone formation. Sclerosis of bone ends.
3 Remodelling	No warmth, swelling, redness, fixed bone deformity, joint stiffness	Appearance of a mature fracture callus. Bony remodelling of major fragments. Decreased sclerosis (rounding of bone ends) signify the finality of the permanent deformity

Laboratory tests included glycosylated haemoglobin (HbA1C) for all research participants. For patients with clinical signs of suspected chronic foot osteomyelitis, total white blood cell count and Erythrocyte sedimentation rate (ESR) were as well requested. HbA1c was considered within the normal range if less than 7%. ESR of more than 70 mm/hr. was considered positive for osteomyelitis [40].

A final diagnosis of Charcot foot arthropathy was made by the PI based on both clinical findings and radiological findings. Three types of conclusion could arise from the examination: Acute CA, Chronic CA, CA associated with osteomyelitis.

Findings leading to confirmation of the diagnosis of acute CA should have all the followings: On observation: foot swelling/oedema/erythema, no single scratch, wound or ulcer. On neurologic examination: reduced or no foot sensation confirmed by a positive 5.07/10 g Semmes-Weinstein monofilament (peripheral neuropathy). On temperature examination: a difference of more than 2 °C with the opposite foot using an infrared dermal thermometer. On foot radiograph: normal radiograph or minimal abnormality different from the one of stages 1 (bony debris, fragmentation, fracture), 2 (coalescence, callus) and 3 (remodeling) of the modified Eichenholtz classification.

Findings leading to confirmation of Chronic CA should have all the followings: On observation: No or reduced swelling/oedema/erythema; no single scratch, wound or ulcer; bone deformity of various degrees. On neurologic examination: reduced or no foot sensation confirmed by a positive 5.07/10 g Semmes-Weinstein monofilament (peripheral neuropathy). On temperature examination: no difference of more than 2 °C with the opposite foot using an infrared dermal thermometer. On foot radiograph: abnormal radiograph showing one of the lesions of stages 1 (bony debris, fragmentation, fracture), 2 (coalescence, callus) or 3 (remodeling) of the modified Eichenholtz classification.

Findings leading to confirmation of the diagnosis of CA associated with osteomyelitis should have all the followings: the presence of a foot ulcer which had been present for more than one week, the lesion extending more than 2cm^2^, and a positive probe to bone test. On neurologic examination: reduced or no foot sensation confirmed by a positive 5.07/10 g Semmes-Weinstein monofilament (peripheral neuropathy). On foot radiograph: abnormal radiograph showing one of the lesions of stages 1 (bony debris, fragmentation, fracture), 2 (coalescence, callus) or 3 (remodeling) of the modified Eichenholtz classification plus other lesions of osteomyelitis. On laboratory investigation: an ESR more than 70 mm/h.

### Data analysis

Data were summarized using descriptive statistics. Bivariate analysis was performed using student t-test for continuous variables and Ranksum Mann Whitney U test categorical variables. A value of less than 0.05 was considered to be associated with CA.

## Results

One hundred adult patients with longstanding diabetes were recruited of which the majority were female (79/100, 79%) and a half was above 50 years of age. Sixty–four patients (64%) were either overweight or obese. Forty-three patients (43%) had suffered from diabetes mellitus for more than 10 years. The mean diabetes duration, mean body mass index and mean HbA1c were respectively 12.8 years (± 5.9 SD), 27.6 (± 5.2 SD) and 6.7% (± 6.7 SD). Patient demographic characteristics are summarized in Table 1.

**Table 1:** Demographic characteristics of research participants

	Mean (± SD)	Frequency (*N* = 100)	Percentage
Age in years	51.3 (12.6)		
Duration of diabetes in years	12.8 (5.9)		
Body Mass Index	27.6 (5.2)		
HbA1c (%)	6.7 (2.4)		
Range of ages in years			
< 30		4.0	4
30–39		9.0	9
40–49		33.0	33
≥ 50		54.0	54
Sex			
Male		21.0	21
Female		79.0	79
Education^a^			
Primary and below		62.0	62
Secondary and above		38.0	38
Body Mass Index			
Underweight		3.0	3
Normal		33.0	33
Overweight		33.0	33
Obese		31.0	31
Diabetes duration			
≤ 10 years		57.0	57
> 10 years		43.0	43
Foot clinical assessment			
History of foot surgery		3.0	3
History of foot trauma		6.0	6
Peripheral neuropathy		27.0	27
Acute Charcot		4.0	4

Education^a^: (Primary = 61, None = 1) and (Secondary = 33, University = 5)

### Clinical and radiological assessment

Twelve patients (12%, 7–20 95% CI) were diagnosed with Charcot foot arthropathy, one-third of which had the acute disease on clinical examination.

Using the Sanders and Frykberg anatomical classification of Charcot foot arthropathy, 2 among the 12 participants with CA had normal radiographs, 5 out of the remaining 10 participants (50%) had type 1 and 5 (50%) had type 3 disease.

**Fig. 1:**
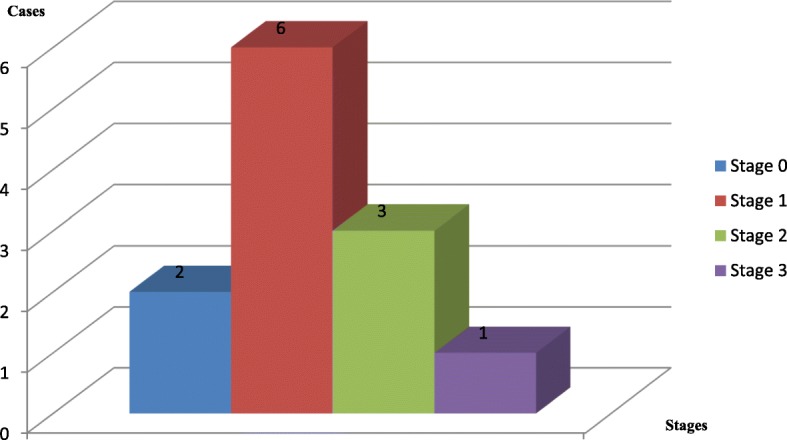
Histogram showing the radiological classification of CA (modified Eichenholtz) among the 12 cases of CA. The different stages (with different colours) are on the x-axis and the number of cases for each stage is expressed on the y-axis and top of each histogram. Radiological classification of Charcot foot arthropathy (modified Eichenholtz) among the 12 patients with CA. 0: Inflammatory phase. 1: Development phase. 2: Healing phase. 3: Remodelling phase. 0 and 1 are early stages, 2 and 3 are late stages

Using the modified Eichenholtz classification, 2 out of 12 participants with CA (17%) had stage 0 lesions, 6 out of 12 participants with CA (50%) had stage 1 lesions, 3 out of 12 participants with CA (25%) had stage 2 lesions, and 1 out of 12 participants with CA (8%) had stage 3 lesions (Fig. 1).

**Fig. 2:**
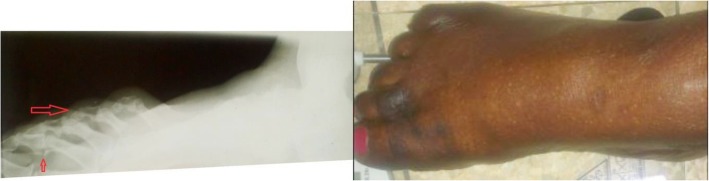
The lateral view foot radiograph and the foot picture of the same patient with CA. The anatomical classification according to Sanders and Frykberg for this foot is type 1 (lesions in forefoot) and 3 (lesions in the tarsal bones). The modified Eichenholtz radiological classification for this foot is stage 1 (foot swelling, fragmentation of subchondral bones in the tarsus, periarticular fracture at the proximal interphalangeal joint of the 2nd toe, toe clawing)

**Fig. 3:**
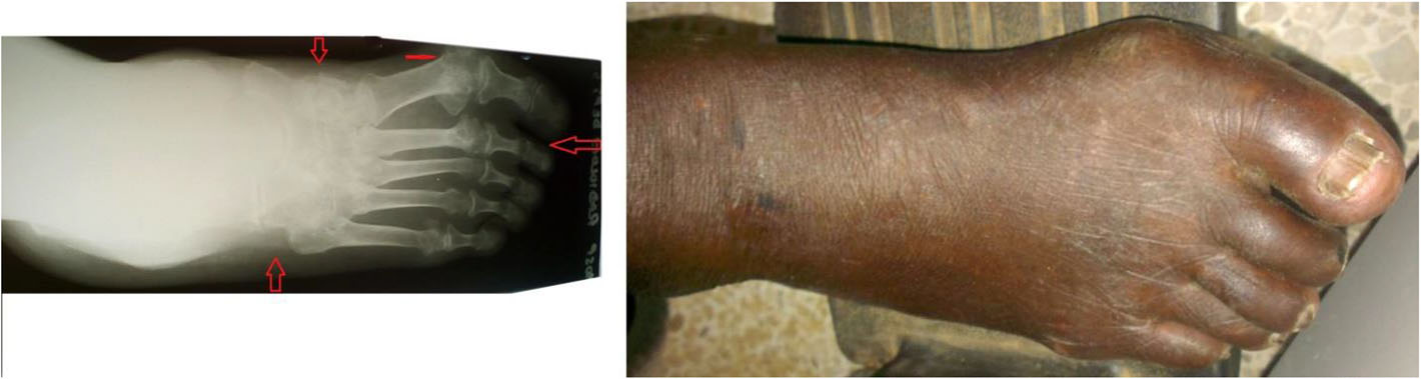
The anteroposterior view foot radiograph and the foot picture of the same patient with CA. The anatomical classification according to Sanders and Frykberg for this foot is type 1 (lesions in forefoot) and 3 (lesions in the tarsal bones). The modified Eichenholtz radiological classification for this foot is stage 3 (callus, bone remodelling at tarsus and metatarsophalangeal joints)

Some feet with CA among the 12 patients and their features on weight-bearing radiographs are illustrated in Figs. 2 and 3.

### Patient factors associated with Charcot foot arthropathy

Bivariate analysis of demographic characteristics showed no association between demographic characteristics (age, BMI, diabetes duration) and CA (Table 2).

**Table 2:** Bivariate analysis of demographic characteristics associated with CA among adult patients with longstanding diabetes

Demographic characteristics	Presence of Charcot *N* = 12	No Charcot *N* = 88
Age in years		
Means ±SD	56.17 ± 11.10	50.59 ± 12.70
95%Confidence interval	(49.11, 63.22)	(47.90, 53.28)
Pr(T > t)	0.1000	0.3589
BMI		
Means ±SD	30.22 ± 6.07	27.24 ± 5.06
95%Confidence interval	(26.36, 34.07)	(26.17, 28.32)
Pr(T > t)	0.0556	0.3183
Diabetes duration years		
Means ±SD	14.25 ± 7.42	12.60 ± 5.71
95%Confidence interval	(9.53, 18.97)	(11.39, 13.81)
Pr(T > t)	0.2188	0.4083

Results are expressed as mean + SD; the Student’s t-test for continuous variables (age, BMI, Duration of diabetes)

Bivariate analysis of clinical and paraclinical characteristics showed that an abnormal foot radiograph was associated with CA (Table 3).

**Table 3:** Bivariate analysis of clinical and paraclinical characteristics associated with CA among adult patients with longstanding diabetes

Clinical / Paraclinical Characteristics	Presence of Charcot *N* = 12 (%)	No Charcot *N* = 88(%)	Prob > |z|
History of foot surgery			
Yes	1 (8)^a^	2 (2)^b^	0.2507
No	11 (92)	86 (98)	
History of foot trauma			
Yes	1 (8)	5 (6)	0.7181
No	11 (92)	83 (94)	
Foot radiograph			
Normal	2 (17)	61 (69)	
Abnormal	10 (83)	27 (31) ^c^	0.0004
HbA1c			
Means ±SD	6.08 ± 2.17	6.74 ± 2.42	
95%Confidence interval	(4.77, 7.40)	(6.23, 7.25)	
Pr(T > t)	0.2121	0.4115	

The Mann-Whitney U-test for categorical variables with unequal variances was used; the Student’s t-test for continuous variables (HbA1c)

^a^The patient had a below knee amputation after foot gangrene ^b^ Patients had below knee amputation after foot gangrene and first toe amputation after big toe gangrene ^c^ Abnormal, no CA: Arthritic changes (19/27, 71%), osteoporosis (6/27, 22%), toe deformities (2/27, 7%, hallux valgus and congenital malformation)

## Discussion

This study aimed to determine the extent, the radiological patterns and associated factors of CA among adult patients with longstanding diabetes mellitus attending an outpatient’s clinic in an African setting.

The majority of participants with CA were in their sixth decade of life. This is in concordance with other studies where CA presents during the fifth and sixth decade [14, 29, 30]. Age is an important risk factor for diabetic neuropathy [46] which is very crucial in the pathogenesis of CA [8, 14, 17, 29, 30].

The proportion of CA among patients with longstanding diabetes in this study was 12%. Several authors have reported varying prevalence rates for CA in different populations ranging from 0.08% to 13 [12, 14, 17, 23, 26]. The prevalence is low when considering the general population with diabetes and increases when considering a specific group among patients with diabetes such as those consulting a foot clinic for a foot problem [14, 17]. Charcot foot arthropathy is not a frequent complication in the general population with diabetes; however, when present it affects the quality of life, threatens the lower limb for amputation and its management is challenging [23, 26]. Routine examination of the feet is very crucial for patients with diabetes because it can avert catastrophic complications. One-third of the participants in this study were in the acute phase of CA. Diagnosis of CA in the acute phase is difficult because clinical features are non-specific and foot radiographs are often normal. Making a diagnosis at this stage reduces morbidity and facilitates proper management [8, 12, 17, 23]. Clinicians should not only have a clear understanding of diabetic foot disease but should also have a high index of suspicion.

In this study, there was an equal distribution of CA in the fore- and mid-foot. This pattern is similar to what has been reported by other authors [14, 23]. In addition to diabetes, the pattern of disease is influenced by other biomechanical factors that act on the foot. For example, a high BMI predisposes the middle arch to collapse culminating in a rocker-bottom foot; tightness of the triceps surae complex leads to abnormal plantar pressure distribution in the forefoot during walking thus exposing the forefoot bones to more strain [8, 17, 23]. Hence, addressing the biomechanics of the foot is also important in the management of these patients [8, 17, 23, 47–49].

Most of the lesions in this study were at the initial stages of the natural history of CA. The mainstay in the treatment of the initial stages (inflammatory and developmental) is non-operative management by offloading the limb in a total contact cast [17, 23, 50]. If these stages are treated judiciously, the achievement of a stable foot without surgery or skin breakdown is possible [17]. This shows the need for quick management intervention in the acute phase before latter stages where bone deformities are fixed and permanent, predisposing the foot to ulceration, infection and high risk of amputation [17]. Clinical symptoms in the inflammatory stage may precede radiographic changes by up to 1 year, hence treatment in this stage is critical in preventing the disease from progressing in further stages [14, 51].

The vast majority of patients in this study had abnormal weight-bearing foot radiographs with no obvious history of trauma. An abnormal weight-bearing foot radiograph in a patient with longstanding diabetes and peripheral neuropathy should raise suspicion of CA. When reading the film one must take time to confirm if the lesions seen are features of CA. Therefore clinicians should have a high index of suspicion and ought to routinely assess patient’s feet for CA.

### Strengths and limitations

This study was based on primary data gathered prospectively, hence allowing us to collect all intended parameters. To our knowledge this study is the first on CA in these settings, giving baseline data for further researches and planning in this field. This study also highlights the utility of corroborating clinical information with simple imaging techniques in making a diagnosis. This is especially important in settings, which only have basic diagnostic equipment; and disease diagnosis requires a high index of suspicion and good clinical acumen.

Results of this study may not be generalizable because of the cross-sectional design, relatively small sample size and the use of a consecutive sampling technique. We did not have access to more advanced imaging modality such as MRI to confirm acute cases of CA with normal foot radiographs.

## Conclusion

Charcot foot arthropathy is a relatively common complication among patients with long-standing diabetes mellitus in our setting. Several cases present in the acute phase when there are no obvious pathological radiographic changes and diagnosis is relatively difficult. This notwithstanding, the majority of patients with a clinical diagnosis of CA may present with abnormal weight-bearing foot radiographs with the involvement of the mid-foot and forefoot. Therefore clinicians should have a high index of suspicion to enable early diagnosis and also prevent potentially disabling complications.

## Data Availability

The datasets used and/or analysed during the current study are available from the corresponding author on reasonable request.

## Abbreviations

CA: Charcot foot arthropathy
ESR: Erythrocyte sedimentation rate
HbA1c: Glycosylated hemoglobin
MNRH: Mulago national referral hospital
